# Decomposing interaction and mediating effects of race/ethnicity and circulating blood levels of cystatin C on cognitive status in the United States health and retirement study

**DOI:** 10.3389/fnhum.2023.1052435

**Published:** 2023-06-01

**Authors:** César Higgins Tejera, Erin B. Ware, Lindsay C. Kobayashi, Mingzhou Fu, Margaret Hicken, Matthew Zawistowski, Bhramar Mukherjee, Kelly M. Bakulski

**Affiliations:** ^1^Department of Epidemiology, School of Public Health, University of Michigan, Ann Arbor, MI, United States; ^2^Institute for Social Research, University of Michigan, Ann Arbor, MI, United States; ^3^Department of Biostatistics, School of Public Health, University of Michigan, Ann Arbor, MI, United States

**Keywords:** cystatin C, disparity, race, mediation, interaction

## Abstract

**Background and objectives:**

Elevated circulating cystatin C is associated with cognitive impairment in non-Hispanic Whites, but its role in racial disparities in dementia is understudied. In a nationally representative sample of older non-Hispanic White, non-Hispanic Black, and Hispanic adults in the United States, we use mediation-interaction analysis to understand how racial disparities in the cystatin C physiological pathway may contribute to racial disparities in prevalent dementia.

**Methods:**

In a pooled cross-sectional sample of the Health and Retirement Study (*n* = 9,923), we employed Poisson regression to estimate prevalence ratios and to test the relationship between elevated cystatin C (>1.24 vs. ≤1.24 mg/L) and impaired cognition, adjusted for demographics, behavioral risk factors, other biomarkers, and chronic conditions. Self-reported racialized social categories were a proxy measure for exposure to racism. We calculated additive interaction measures and conducted four-way mediation-interaction decomposition analysis to test the moderating effect of race/ethnicity and mediating effect of cystatin C on the racial disparity.

**Results:**

Overall, elevated cystatin C was associated with dementia (prevalence ratio [PR] = 1.2; 95% CI: 1.0, 1.5). Among non-Hispanic Black relative to non-Hispanic White participants, the relative excess risk due to interaction was 0.7 (95% CI: −0.1, 2.4), the attributable proportion was 0.1 (95% CI: −0.2, 0.4), and the synergy index was 1.1 (95% CI: 0.8, 1.8) in a fully adjusted model. Elevated cystatin C was estimated to account for 2% (95% CI: −0, 4%) for the racial disparity in prevalent dementia, and the interaction accounted for 8% (95% CI: −5, 22%). Analyses for Hispanic relative to non-white participants suggested moderation by race/ethnicity, but not mediation.

**Discussion:**

Elevated cystatin C was associated with dementia prevalence. Our mediation-interaction decomposition analysis suggested that the effect of elevated cystatin C on the racial disparity might be moderated by race/ethnicity, which indicates that the racialization process affects not only the distribution of circulating cystatin C across minoritized racial groups, but also the strength of association between the biomarker and dementia prevalence. These results provide evidence that cystatin C is associated with adverse brain health and this effect is larger than expected for individuals racialized as minorities had they been racialized and treated as non-Hispanic White.

## Introduction

Racial disparities in cognitive aging are well-documented (Chin et al., 2011), and thought to derive from exposure to structural and interpersonal forms of racism across the life course, including but not limited to residential and educational segregation (Mantri et al., 2019; Caunca et al., 2020), socioeconomic barriers (Glymour and Manly, 2008), and psychosocial stress and discrimination (Geronimus et al., 2006). In the United States, minoritized and racialized African Americans have two times higher odds of prevalent dementia and Hispanics have 1.5 times higher odds of prevalent dementia than White Americans (Chen and Zissimopoulos, 2018). Research has shown that physiological systems may be affected in minoritized social groups exposed to racism (Harrell et al., 2003). Interpersonal forms of racism, such as perceived discrimination, have been associated with poor cognitive development (Seaton, 2010) and a moderate decline in kidney function (Beydoun et al., 2017). The link between chronic kidney disease and adverse cognitive outcomes has been documented through several studies (Sarnak et al., 2008; Sundelöf et al., 2008; Yaffe et al., 2008; Slinin et al., 2015) and meta-analyses (Berger et al., 2012; Masson et al., 2015). Minoritized groups in the United States are at increased risk for both adverse renal (Nicholas et al., 2013) and cognitive outcomes (Chin et al., 2011; Mayeda et al., 2016; Chen and Zissimopoulos, 2018). Understanding the share of cognitive impairment related to renal dysfunction across racial/ethnic groups may contribute to the identification of target areas for ameliorating racial disparities in cognitive outcomes.

Cystatin C is an inhibitor of cysteine protease activity and cathepsins (Turk et al., 2000) and an important surrogate of kidney function (Peralta et al., 2011a,b). In the central nervous system, cystatin C colocalizes with β-amyloid plaques and cystatin C has neuroprotective properties, inspiring research into its association with Alzheimer’s disease and other dementias (Gauthier et al., 2011; Kaur and Levy, 2012). At least four mechanisms that support the neuroprotective hypothesis have been proposed. First, cystatin C may induce neuroprotection by antagonizing the effect of cysteine proteases (Gauthier et al., 2011; Kaur and Levy, 2012). Second, cystatin C promotes autophagy in cells exposed to oxidative stress and nutritional deprivation (Gauthier et al., 2011; Kaur and Levy, 2012). Third, cystatin C may induce neuroprotection by inhibiting Aβ-amyloid fibril formation (Gauthier et al., 2011; Kaur and Levy, 2012). Finally, cystatin C bolsters cell proliferation in areas of the brain exposed to injury (Gauthier et al., 2011; Kaur and Levy, 2012). However, studies measuring cystatin C in cerebrospinal fluid are costly and usually conducted in small samples. Because of these limitations, epidemiological studies have focused attention on the association between serum circulating cystatin C levels and cognitive outcomes (Harrell et al., 2003; Seaton, 2010; Chen and Zissimopoulos, 2018). The mechanisms by which peripheral levels of cystatin C relate to adverse cognitive aging are less clear. But epidemiological evidence suggests that higher serum levels of cystatin C correlate with atherosclerotic plaque burden of the coronary arteries, which may play an important role in the genesis of cardiovascular and cerebrovascular events and underscore the active role of cystatin C in brain health (Madero et al., 2009; O’Seaghdha et al., 2014; Chen et al., 2021). Serum cystatin C levels are an alternative biomarker of kidney function to that of creatinine-based measures because they are less susceptible to the influence of body mass index, age, sex, and diet (Inker et al., 2012). Diverse studies have linked cystatin C to chronic conditions such as end stage renal disease (Mantri et al., 2019), cardiovascular disease (Madero et al., 2009; McMurray et al., 2009; O’Seaghdha et al., 2014), and more recently impaired cognition. The increasing body of evidence linking high circulating levels of cystatin C to cerebrovascular outcomes suggest that cystatin C may play an important role in the development of atherosclerosis and cerebrovascular disease, with evidence pointing at shared biological processes such as oxidative stress, micro-vascular inflammation, and plaque formation that are present in both early stages renal disease and vascular forms of dementia (Madero et al., 2009; O’Seaghdha et al., 2014; Chen et al., 2021, 2022). In large population studies, circulating cystatin C varies by race/ethnicity. For example, a study found that in adults of age 50−59, non-Hispanic Black had 2.23 and Mexican Americans had 1.01 higher odds of elevated serum cystatin C levels (i.e., >1.12 mg/L) than their non-Hispanic White counterparts, even after adjustment for known risk factors of chronic kidney disease (Köttgen et al., 2008). When the same analysis was conducted among adults of age 60 or more, non-Hispanic Black had 0.74 and Mexican Americans had 0.53 lower odds of elevated serum cystatin C levels. Researchers argued that the observed discrepancy between racialized social groups and elevated cystatin C in age stratified analysis may obey to selection bias issues and differential survival between compared groups (Köttgen et al., 2008). However, prior studies of cystatin C and cognitive outcomes in older adults have not explored race-dependent relationships (Sarnak et al., 2008; Sundelöf et al., 2008; Yaffe et al., 2008; Slinin et al., 2015)—addressing this gap is essential for three main reasons. First, identifying subpopulations at most risk for dementia may provide insights for the highest impact interventions. Second, quantifying the mediating effect of cystatin C on the relationships between race/ethnicity and dementia may identify biological pathways underlying racial and ethnic disparities in cognition. Third, estimating the proportion of racial and ethnic disparities in dementia that could be reduced if cystatin C was amenable to intervention is useful to inform public health efforts and policies.

In a nationally representative racially/ethnically diverse sample of middle-aged and older adults in the United States, we tested the associations between circulating cystatin C levels and cognitive status. Using four-way mediation-interaction decomposition analysis (VanderWeele, 2014), we aimed to examine the moderating effect of race/ethnicity in the association between cystatin C and cognitive status, and to understand how elevated levels of cystatin C may be a pathway (mediator) linking racial and ethnic disparities in cognitive impairment.

## Materials and methods

### Study population

The United States Health and Retirement Study is an ongoing longitudinal study consisting of a representative sample of adults in the United States over age 50 (Juster and Suzman, 1995). To ensure representativeness of the United States population, recruitment is based on a multi-stage probability design with oversampling of underrepresented demographic groups in research (non-Hispanic Black and Hispanic participants). The initial cohort was formed in 1992 and interviews are conducted in 2-year waves (Sonnega et al., 2014). Primary respondents who meet age-eligibility criteria (age 50 +) are randomly selected for interview along with a partner in the household, regardless of the partner’s age. For this analysis, we pooled a cross-sectional sample of participants who provided a blood spot at either the 2006 or 2008 wave. Proxy respondents were excluded, as they did not provide blood spots. Participants provided written informed consent at the time of the data collection. These secondary data analyses were approved by the University of Michigan Institutional Review Board (HUM00128220). Survey data are publicly available,^1^ and genetic data are available through dbGaP^2^.

### Race and ethnicity

In the HRS, race is documented by self-reports across different waves of data. Study participants are asked to select their primary race from three large categories White/Caucasian, Black/African Americans, or Other. In an additional question, participants indicate whether they also identified as Hispanic or Latino. From these responses, we created a three mutually exclusive categorial variable (i.e., non-Hispanic Black, Hispanic, and non-Hispanic White categories). We used participants’ self-reported racialized category as a proxy indicator of exposure to racism and we compared cognitive status on each minoritized racial and ethnic category (non-Hispanic Black and Hispanic) to non-Hispanic White participants.

### Cystatin C

Dried blood spots were collected on a randomly selected half of the HRS cohort in the 2006 wave and the other half of the cohort in 2008. Levels of cystatin C were measured in blood spots using an optimized ELISA protocol (Crimmins et al., 2014). Assay performance was standardized to the European Certified Reference Material for cystatin C with Human Serum ERM-DA471/IFCC. Test results were verified by comparing 139 dried blood spot samples to matched plasma samples analyzed by microtiter plate ELISA. ELISA cystatin C levels were normalized to the United States National Health and Nutrition Examination Survey to ensure measures were comparable to venous blood and representative of the United States adult population. We considered quartiles of cystatin C in our exploratory analysis; and dichotomized cystatin C at the 75th percentile (1.24 mg/L) in our regression models, this cutoff point was used in a previous research study (Yaffe et al., 2008). Participants with cystatin C levels greater than 1.24 mg/L were considered high, and those less than or equal to 1.24 mg/L were considered low.

To represent the additive interactions between elevated cystatin C levels and race/ethnicity, we operationalized cystatin C and each of race and ethnicity together [1 = non-Hispanic White participants with low cystatin C (unexposed group), 2 = non-Hispanic White participants with high cystatin C, 3 = non-Hispanic Black participants with low cystatin C, 4 = non-Hispanic Black participants with high cystatin C (double exposed)]. We refer to the second and third levels as the “single-exposed” groups, because they have only one of the two exposures (minoritized race/ethnicity status or high cystatin C). We created a similar four-level variable for Hispanic participants, using non-Hispanic White participants with low cystatin C as the reference group. This operationalization allowed us to estimate the joint effect of race/ethnicity and cystatin C on cognitive function and directly estimate measures of interaction in the additive scale (Assmann et al., 1996).

### Cognitive assessment

Cognitive function was assessed by the Telephone Interview Cognitive Status, which provides a total score of 0−27 points based on a battery of cognitive tests assessing immediate and delayed recall of 10 words, serial 7s, counting backward, object naming, and recalling the date, president, and vice-president to assess orientation (Crimmins et al., 2011). We used the Langa-Weir algorithm to classify cognitive function into three mutually exclusive categories: normal cognition (12−27), cognitive impairment non-dementia (CIND) (7−11), and dementia (0−6) (Crimmins et al., 2011).

### Covariate measures

Relevant covariates and known confounding factors between circulating cystatin C and cognitive status were identified from previous studies (Sarnak et al., 2008; Yaffe et al., 2008; Slinin et al., 2015). Covariates were self-reported in the HRS interviews, and were: age (continuous, in years, calculated from birth date and interview date), sex (male; female), educational attainment (high school or less; some college, college, or more than college), number of alcoholic drinks per day (continuous), smoking status (current smoker; former smoker; never smoker), body mass index (kg/meters^2^, continuous, based on self-reported height and weight), exercise was operationalized as a dichotomous variable (Yes; No) to capture whether a participant engaged in vigorous, moderate, or light physical activity at least >1 time per week. Sleep disturbance (Yes; No) captures whether a participant had restless sleep (Yes; No), and number of chronic health conditions included high blood pressure (yes or no), diabetes (yes or no), cancer (yes or no), lung disease (yes or no), heart disease (yes or no), stroke (yes or no), psychiatric problems (yes or no), and arthritis (yes or no), and was operationalized in our models as continuous (0−8 conditions).

### Biomarkers

Other biomarkers measured in the dried blood spots were included as covariates (Crimmins et al., 2014). Levels of total cholesterol and high-density lipoprotein cholesterol were measured by microtiter plate assay using conventional clinical chemistry reactions optimized for the limited volume available from a dried blood spot sample. We constructed a ratio of total cholesterol to high density lipoprotein cholesterol as a marker of atherosclerosis. Levels of glycosylated hemoglobin (HbA1C) were measured on the dried blood spot; the assay was performed using Bio-Rad Laboratories Variant II High Pressure Liquid Chromatography (HPLC) System (Hercules, CA, USA) optimized to accommodate the limited volume. We used HbA1C in is continuous form as a maker of glucose homeostasis.

### Genetic marker

Carriers of the Apolipoprotein E ε*4* allele (*APOE*-ε4) are at increased risk of cognitive impairment (Liu et al., 2013, 2017). Information on *APOE*-ε*4* allele carrier status was obtained from genetic data imputed to the worldwide 1,000 Genomes Project reference panel. Dosage data for rs7412 and rs429358 was used to determine ε*4* carrier status (Faul et al., 2021). We dichotomized the *APOE*-ε*4* carrier status into respondents with at least 1 copy of the allele ε*4* vs. none and used carrier status in sensitivity models. Genotyping and imputation information on the Health and Retirement Study is available elsewhere (Genetic Data, 2022).

### Statistical analyses

We restricted our sample to participants with complete data for all covariates of interest. We described the bivariate relationships between race/ethnicity and our covariates of interest using chi-square tests for categorical variables and one-way ANOVA tests for continuous covariates. We described the quartiles of circulating cystatin C and categorical covariates. We also described the distributions of covariates according to the combined cystatin C (high vs. low) and race/ethnicity categories. Because cystatin C is a marker of renal function (Peralta et al., 2011b) and kidney performance declines with age, (Köttgen et al., 2008) we calculated age-group-specific (e.g., less than 60 years old, 60−79, 70−79, or 80 and more) predicted probabilities of high cystatin C (i.e., >1.24 mg/L) for each racial/ethnic group. We plotted marginal predicted probabilities of high cystatin C (dependent variable) vs. age (explanatory variable) to understand whether concentrations of elevated cystatin C varied across the three racialized group at different decades of life.

We used multivariable-adjusted Poisson regression models with a logarithmic link function and robust error variance (Zou, 2004) to estimate prevalence ratios for the relationships between high cystatin C levels and each of CIND and dementia relative to normal cognition in the overall study sample and stratified by each racial group. We estimated four regression models: Model 1 was unadjusted, Model 2 was adjusted for demographic variables (age, sex, education), Model 3 was additionally adjusted for behavioral risk factors (smoking status, alcohol consumption, body mass index, exercise, and sleep), and Model 4 was additionally adjusted for two biomarkers (the ratio of total cholesterol to high density lipoprotein cholesterol, HbA1C) and chronic conditions. We calculated three measures of interaction in the additive scale, the relative excess risk due to interaction, the proportion attributable to the interaction, and the synergy index to evaluate effect modification of the association between cystatin C and prevalent dementia by racialized social groups (Assmann et al., 1996). We performed four-way mediation-interaction decomposition analysis to evaluate whether cystatin C was a mediator of the racial disparity while allowing the mediator to interact with the exposure variable race/ethnicity (VanderWeele, 2014). We illustrated the relationship between exposure, mediator, outcome, and confounding variables in a directed acyclic graph (Supplementary Figure 1). Detailed information on how interaction measures and the four-way mediation-interaction decomposition analysis were calculated can be found in the Supplementary material.

### Sensitivity analyses

*APOE*-ε*4* was not available for the full study sample. Thus, we tested *APOE*-ε*4* carrier status in a sensitivity analysis where the genetic marker was added to our previously specified demographic models (Model 2). These models were stratified by race/ethnicity. Because the Langa-Weir algorithm may have differential sensitivity across racial/ethnic groups, we also assessed dementia using the Power’s dementia algorithm (dementia vs. normal cognition), a recently developed algorithm with comparable sensitivity of dementia classification across HRS racial groups (Gianattasio et al., 2020).

Analyses were conducted using R statistical software (version 3.6.2) and Stata (v14). For reproducibility purposes, we compared the code we developed to the Stata macro med4way (Discacciati et al., 2019). A second analyst performed complete code review. Code to produce these analyses is available^3^.

## Results

### Sample characteristics

There were 9,923 participants included in our analytic sample (Supplementary Figure 2). The sample had an average age of 68.4 years, with 12.6 years of education, and 1.12 mg/L circulating cystatin C levels (Table 1). In this sample, 13% of participants were non-Hispanic Black, 9% were Hispanic, 16% had cognitive impairment non-dementia, and approximately 4% had dementia. The prevalence of dementia was higher among non-Hispanic Black (12%) and Hispanic (9%) adults than non-Hispanic White (3%) adults (Table 1). Average circulating levels of cystatin C were also higher for non-Hispanic Black participants (1.18 mg/L), but comparable levels were observed for Hispanic (1.11 mg/L) and non-Hispanic White (1.11 mg/L) participants (Table 1). Excluded participants were slightly more likely to have CIND or dementia than included participants, but did not differ in their distributions of race, education, alcohol consumption, smoking status, sleep disturbance and chronic conditions (Supplementary Table 1). Excluded participants had lower concentrations of cystatin C to included participants, higher baseline HbA1C, and higher total cholesterol to high density lipoprotein cholesterol ratio.

**TABLE 1:** Sample characteristics by racialized social groups in the United States health and retirement study, 2006 and 2008 waves.

Characteristic	Overall *N* = 9,923^1^	Non-Hispanic black *N* = 1,268^1^	Hispanic *N* = 920^1^	Non-Hispanic white *N* = 7,735^1^	*p*-value^2^
Cystatin C (mg/L)	1.12 (0.54)	1.18 (0.80)	1.11 (0.72)	1.11 (0.46)	**<0.001**
Dementia status					**<0.001**
Cognitively normal	8,019 (96%)	771 (88%)	619 (91%)	6,629 (97%)	
Dementia	345 (4.1%)	105 (12%)	58 (8.6%)	182 (2.7%)	
Impairment status					**<0.001**
Cognitively normal	8,019 (84%)	771 (66%)	619 (72%)	6,629 (88%)	
Cognitive impairment non-dementia	1,559 (16%)	392 (34%)	243 (28%)	924 (12%)	
Gender					**<0.001**
Female	5,961 (60%)	830 (65%)	586 (64%)	4,545 (59%)	
Male	3,962 (40%)	438 (35%)	334 (36%)	3,190 (41%)	
Education					**<0.001**
College/some or >	2,590 (26%)	212 (17%)	93 (10%)	2,285 (30%)	
HS or <	7,333 (74%)	1,056 (83%)	827 (90%)	5,450 (70%)	
Age (years)	68.38 (10.45)	67.25 (9.90)	65.15 (10.48)	68.95 (10.45)	**<0.001**
Years of education	12.58 (3.12)	11.68 (3.14)	9.15 (4.52)	13.13 (2.56)	**<0.001**
Alcohol (# drinks/day when drinks)	0.69 (1.40)	0.50 (1.28)	0.61 (1.99)	0.73 (1.33)	**<0.001**
Smoking status					**<0.001**
Current smoker	1,371 (14%)	227 (18%)	116 (13%)	1,028 (13%)	
Former smoker	4,280 (43%)	503 (40%)	358 (39%)	3,419 (44%)	
Never smoker	4,272 (43%)	538 (42%)	446 (48%)	3,288 (43%)	
Body mass index (kg/m^2^)	28.29 (5.86)	30.06 (6.64)	29.22 (5.65)	27.89 (5.68)	**<0.001**
Exercise					**<0.001**
Yes	7,707 (78%)	868 (68%)	697 (76%)	6,142 (79%)	
No	2,216 (22%)	400 (32%)	223 (24%)	1,593 (21%)	
Sleep					**<0.001**
Yes	3,065 (31%)	381 (30%)	339 (37%)	2,345 (30%)	
No	6,858 (69%)	887 (70%)	581 (63%)	5,390 (70%)	
Chronic conditions	2.04 (1.43)	2.30 (1.49)	1.81 (1.40)	2.02 (1.42)	**<0.001**
Baseline A1C (%)	5.86 (0.97)	6.21 (1.22)	6.23 (1.41)	5.76 (0.83)	**<0.001**
Baseline TC/HDL-C (mg/dL)	3.97 (1.19)	3.85 (1.14)	4.12 (1.24)	3.97 (1.19)	**<0.001**

^1^Statistics presented: N (Col%); Mean (SD). ^2^Statistical tests performed: chi-square test of independence; one-way ANOVA. TC/HDL-C: total cholesterol to high density lipoprotein-cholesterol ratio; HbA1C: glycosylated hemoglobin. Values in bold are statistically significant.

### Associations between cystatin C and cognitive status

Among those with cystatin C >1.24 mg/L, the prevalence of CIND was 25%, and among those with levels <0.86 mg/L was 11% (Supplementary Table 2). Among those with cystatin C levels >1.24 mg/L, the prevalence of dementia was 8.7%, and among those with levels <0.86 mg/L was 1.9% (Supplementary Table 2 and Supplementary Figure 3). Participants with cystatin C >1.24 mg/L were more likely to be non-Hispanic White, to have high school education or less, and to be older than those with <0.86 mg/L cystatin C levels (Supplementary Table 2). Among participants in their 50 s through 70 s, the non-Hispanic Black group had higher cystatin C than the other two racial/ethnic groups (Supplementary Figure 4). Across all ages, the prevalence of high cystatin C was similar for the non-Hispanic White and Hispanic groups (Supplementary Figure 4).

**FIGURE 1:**
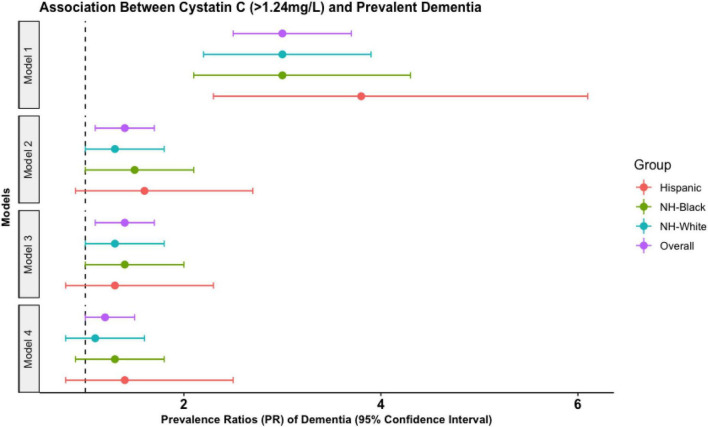
Prevalence ratio (PR) estimates from Poisson regression models illustrating the association between elevated cystatin C levels (>1.24 mg/L) and prevalent dementia in the United States Health and retirement study waves 2006 and 2008. Estimates are derived from the overall sample and by each racialized social group. NH-black, non-Hispanic black participants; NH-white, non-Hispanic white participants. Model 1 (unadjusted). Model 2 (demographic): adjusted for age, sex, education. Model 3 (behavioral): adjusted for demographics, smoking status, alcohol consumption, body mass index, exercise, sleep. Model 4 (biomarker model): adjusted for behavioral factors, chronic conditions, total cholesterol to high density lipoprotein-cholesterol ratio, glycosylated hemoglobin.

In our overall sample, model 4 showed that those with high cystatin C had 1.2 (95% CI: 1.0, 1.5) times higher prevalence of dementia and 1.1 (95% CI: 1.0, 1.3) times higher prevalence of CIND than those with low cystatin C levels (Figure 1 and Table 2). The association between high cystatin C and dementia—but not cognitive impairment non-dementia—was similar across all racial groups (Figure 1 and Table 2).

**TABLE 2:** Adjusted prevalence ratios between high cystatin C levels (>1.24 mg/L) and dementia, and cognitive impairment non-dementia in the United States health and retirement study stratified by race/ethnicity, waves 2006 and 2008.

	Dementia							
	Overall^1^		Non-Hispanic white		Non-Hispanic black		Hispanic	
**Cystatin C, (mg/L)**								
**Unadjusted**	**PR** ^2^	**95% CI** ^2^	**PR** ^2^	**95% CI** ^2^	**PR** ^2^	**95% CI** ^2^	**PR** ^2^	**95% CI** ^2^
<75th (Reference)	Ref	−	Ref	−	Ref	−	Ref	−
75th (>1.24)	3.0***	[2.5, 3.7]	3.0***	[2.2, 3.9]	3.0***	[2.1, 4.3]	3.8***	[2.3, 6.1]
**Demographic** ^+^								
<75th (Reference)	Ref	−	Ref	−	Ref	−	Ref	−
75th (>1.24)	1.4**	[1.1, 1.7]	1.3	[1.0, 1.8]	1.5*	[1.0, 2.1]	1.6	[0.9, 2.7]
**Behavioral** ^$^								
<75th (Reference)	Ref	−	Ref	−	Ref	−	Ref	−
75th (>1.24)	1.4**	[1.1, 1.7]	1.3	[1.0, 1.8]	1.4*	[1.0, 2.0]	1.3	[0.8, 2.3]
**Biomarkers** ^§^								
<75th (Reference)	Ref	−	Ref	−	Ref	−	Ref	−
75th (>1.24)	1.2*	[1.0, 1.5]	1.1	[0.8, 1.6]	1.3	[0.9, 1.8]	1.4	[0.8, 2.5]
**Cognitive impairment non-dementia**								
**Unadjusted**								
<75th (Reference)	Ref	−	Ref	−	Ref	−	Ref	−
75th (>1.24)	1.8***	[1.7, 2.0]	2.0***	[1.8, 2.3]	1.7***	[1.4, 2.0]	1.4**	[1.1, 1.8]
**Demographic** ^+^								
<75th (Reference)	Ref	−	Ref	−	Ref	−	Ref	−
75th (>1.24)	1.2***	[1.1, 1.4]	1.2**	[1.1, 1.4]	1.3***	[1.1, 1.6]	1.0	[0.8, 1.3]
**Behavioral** ^$^								
<75th (Reference)	Ref	−	Ref	−	Ref	−	Ref	−
75th (>1.24)	1.2***	[1.1, 1.3]	1.2*	[1.0, 1.3]	1.3***	[1.1, 1.6]	1.0	[0.8, 1.3]
**Biomarkers** ^§^								
<75th (Reference)	Ref	−	Ref	−	Ref	−	Ref	−
75th (>1.24)	1.1**	[1.0, 1.3]	1.1	[1.0, 1.2]	1.3**	[1.1, 1.5]	1.0	[0.8, 1.3]

^+^Demographic model: adjusted for age, sex, education. ^$^Behavioral model: adjusted for age, sex, education, smoking status, alcohol consumption, body mass index, exercise, sleep. ^§^ Biomarker model: adjusted for age, sex, education, smoking status, alcohol consumption, body mass index, exercise, sleep, chronic conditions, total cholesterol to high density lipoprotein-cholesterol ratio, glycosylated hemoglobin. ^1^Overall model additionally adjust for race (reference group used for comparison: non-Hispanic white participants). ^2^PR, prevalence ratio; CI, confidence interval in brackets; Ref, reference category used for comparison. *p < 0.05, **p < 0.01, ***p < 0.001.

### Joint associations between race/ethnicity and high cystatin C with cognitive status

Dementia was more prevalent among those who were non-Hispanic Black with high cystatin C (25% in this double exposed group) than those who were non-Hispanic Black with low cystatin C (8.2% in this single exposed group), non-Hispanic White with high cystatin C (5.5% in this single exposed group), or non-Hispanic White with low cystatin C (1.9% in this unexposed group; Supplementary Table 3). Similar patterns were observed for CIND. For Hispanic participants with high cystatin C levels (double exposed group), the prevalence of dementia and CIND was higher than Hispanic participants with low cystatin C (single exposed group), non-Hispanic White with high cystatin C (single exposed group), and non-Hispanic White with low cystatin C (unexposed group) (Supplementary Table 4).

In multivariable-adjusted models, we found that the double exposed group (non-Hispanic Black participants with high cystatin C) had higher prevalence of dementia than the unexposed group (non-Hispanic White participants with low cystatin C) (Table 3). Model 4 showed non-Hispanic Black participants with high cystatin C levels (double exposed group) had 5.8 (95% CI: 4.3, 7.9) times the prevalence of dementia than non-Hispanic White participants with low cystatin C (unexposed group) (Table 3). Similar findings were observed for Hispanic participants relative to non-Hispanic White participants. Model 4 showed that Hispanic participants with high cystatin C levels (double exposed group) had 5.0 (95% CI: 3.3, 7.5) times the prevalence of dementia than non-Hispanic White participants with low cystatin C (unexposed group) (Table 3). In general, for both minoritized racial groups, the associations between the double exposed compared to single exposed and unexposed groups in the CIND models were in the same direction but of lesser magnitudes to those observed in the dementia models (Table 3). However, in the Hispanic sample these associations were attenuated compared to the non-Hispanic Black sample.

**TABLE 3:** Prevalence ratios estimates from Poisson regression models illustrating the association between the joint effect of race/ethnicity and high cystatin C serum levels (>1.24 mg/L) and dementia or cognitively impaired non-dementia in the health and retirement study, waves 2006 and 2008.

	Non-Hispanic Black						Hispanic					
	Dementia			Cognitively impaired non-dementia			Dementia			Cognitively impaired non-dementia		
**Models**	**PR** ^1^	**95% CI** ^1^	***p*-value**	**PR** ^1^	**95% CI** ^1^	***p*-value**	**PR** ^1^	**95% CI** ^1^	***p*-value**	**PR** ^1^	**95% CI** ^1^	***p*-value**
**Unadjusted**												
Unexposed^a^	Ref	—		Ref	—		Ref	—		Ref	—	
Single exposed^b^ (CysC)	3	[2.2, 3.9]	**< *0*.001**	2	[1.8, 2.3]	**< *0*.001**	3	[2.2, 3.9]	**< *0*.001**	2	[1.8, 2.3]	**< *0*.001**
Single exposed^c^ (minority)	4.4	[3.2, 6.0]	**< *0*.001**	2.9	[2.6, 3.3]	**< *0*.001**	3.1	[2.1, 4.6]	**< *0*.001**	2.7	[2.3, 3.1]	**< *0*.001**
Double exposed^d^	13.3	[9.8, 18.2]	**< *0*.001**	4.9	[4.3, 5.7]	**< *0*.001**	11.8	[7.9, 17.5]	**< *0*.001**	3.8	[3.0, 4.7]	**< *0*.001**
**Demographic** ^+^												
Unexposed^a^	Ref	—		Ref	—		Ref	—		Ref	—	
Single exposed^b^ (CysC)	1.4	[1.0, 1.8]	**< *0*.05**	1.3	[1.1, 1.5]	**< *0*.001**	1.3	[1.0, 1.8]	0.05	1.3	[1.1, 1.4]	**< *0*.001**
Single exposed^c^ (minority)	5	[3.7, 6.8]	**< *0*.001**	3	[2.7, 3.4]	**< *0*.001**	3.9	[2.7, 5.6]	**< *0*.001**	2.9	[2.5, 3.3]	**< *0*.001**
Double exposed^d^	7	[5.2, 9.3]	**< *0*.001**	3.7	[3.2, 4.2]	**< *0*.001**	5.8	[3.9, 8.6]	**< *0*.001**	2.5	[2.0, 3.1]	**< *0*.001**
**Behavioral** ^$^												
Unexposed^a^	Ref	—		Ref	—		Ref	—		Ref	—	
Single exposed^b^ (CysC)	1.4	[1.0, 1.8]	**< *0*.05**	1.2	[1.1, 1.4]	**< *0*.001**	1.3	[1.0, 1.8]	> *0*.05	1.2	[1.1, 1.4]	**< *0*.01**
Single exposed^c^ (minority)	5	[3.7, 6.8]	**< *0*.001**	3	[2.7, 3.4]	**< *0*.001**	3.8	[2.6, 5.5]	**< *0*.001**	2.8	[2.4, 3.2]	**< *0*.001**
Double exposed^d^	6.4	[4.7, 8.7]	**< *0*.001**	3.5	[3.0, 4.1]	**< *0*.001**	5.7	[3.8, 8.5]	**< *0*.001**	2.5	[2.0, 3.1]	**< *0*.001**
**Biomarkers** ^§^												
Unexposed^a^	Ref	—		Ref	—		Ref	—		Ref	—	
Single exposed^b^ (CysC)	1.2	[0.9, 1.6]	> *0*.05	1.2	[1.0, 1.3]	**< *0*.001**	1.2	[0.9, 1.6]	> *0*.05	1.1	[1.0, 1.3]	**< *0*.05**
Single exposed^c^ (minority)	4.9	[3.6, 6.7]	**< *0*.001**	3	[2.6, 3.4]	**< *0*.001**	3.7	[2.5, 5.3]	**< *0*.001**	2.8	[2.4, 3.2]	**< *0*.001**
Double exposed^d^	5.8	[4.3, 7.9]	**< *0*.001**	3.3	[2.8, 3.8]	**< *0*.001**	5	[3.3, 7.5]	**< *0*.001**	2.4	[1.9, 2.9]	**< *0*.001**
Observations	7,687			8,716			7,488			8,415		

^1^PR, prevalence ratio; CI, confidence interval; CysC, cystatin C; Ref, reference category. *^a^*Unexposed group (reference group): non-Hispanic white and low cystatin C (<1.24 mg/L). *^b^*Single exposed (CysC): non-Hispanic white and high cystatin C (>1.24 mg/L). *^c^*Single exposed (minority): either non-Hispanic black or Hispanic and low cystatin C (<1.24 mg/L). *^d^*Double exposed: either non-Hispanic Black or Hispanic and High cystatin C (>1.24 mg/L). ^+^Demographic model: adjusted for age, sex, education. ^$^Behavioral model: adjusted for age, sex, education, smoking status, alcohol consumption, body mass index, exercise, sleep. ^§^ Biomarker model: adjusted for age, sex, education, smoking status, alcohol consumption, body mass index, exercise, sleep, chronic conditions, total cholesterol to high density lipoprotein-cholesterol ratio, glycosylated hemoglobin. Values in bold are statistically significant.

#### Measures of interaction on the additive scale between race/ethnicity, cystatin C, and dementia prevalence

Among non-Hispanic Black participants, model 4 yielded a relative excess risk due to interaction of 0.7 (95% CI: −1.0, 2.4), indicating that the joint effect of having a double exposure exceeded the simple addition of being either a non-Hispanic Black participant or having high cystatin C (Table 4). Additionally, from the same model 4 we estimated an attributable proportion of 0.1 (95% CI: −0.2, 0.4), which suggests that 10% of the relative excess risk of dementia in the double exposed group may be attributable to the interaction between the two main exposures (race/ethnicity and high cystatin C). The synergy index represents the quotient between the prevalence ratio of dementia in the double exposed group divided by the isolated prevalence ratios of each single exposed group, a quotient greater than one indicates possible synergistic effect or effect modification. In model 4, the synergy index was 1.1 (95% CI: 0.8, 1.8), indicating potential synergism between both exposures. We obtained estimates of greater magnitude for the other unadjusted and demographically adjusted models, suggesting that behavioral factors, chronic conditions, and other biomarkers may be important confounding variables.

**TABLE 4:** Measures of interaction in the additive scale of the joint effect of race/ethnicity and high cystatin C (>1.24 mg/L) on dementia.

	Non-Hispanic Black^a^			Hispanic^a^		
Models	Estimate	95% CI^1^	*p*-value	Estimate	95% CI^1^	*p*-value
**Unadjusted**						
Excess risk due to interaction^†^	7.0	[3.3, 10.6]	**<0.001**	6.7	[2.3, 11.1]	**<0.01**
Attributable proportion^‡^	0.5	[0.4, 0.7]	**<0.001**	0.6	[0.4, 0.8]	**<0.001**
Synergy index^£^	2.3	[1.6, 3.3]	**<0.001**	2.6	[1.4, 3.9]	**<0.001**
**Demographic** ^+^						
Excess risk due to interaction^†^	1.6	[−0.4, 3.5]	0.11	1.6	[−0.8, 4.0]	0.18
Attributable proportion^‡^	0.2	[−0.0, 0.5]	0.06	0.3	[−0.1, 0.6]	0.10
Synergy index^£^	1.4	[0.9, 2.0]	0.11	1.5	[0.8, 2.7]	0.17
**Behavioral** ^$^						
Excess risk due to interaction^†^	1.0	[−0.9, 2.9]	0.30	1.5	[−0.8, 3.8]	0.19
Attributable proportion^‡^	0.2	[−0.1, 0.4]	0.24	0.3	[−0.1, 0.6]	0.11
Synergy index^£^	1.2	[0.8, 1.8]	0.28	1.5	[0.8, 2.7]	0.17
**Biomarkers** ^§^						
Excess risk due to interaction^†^	0.7	[−1.0, 2.4]	0.42	1.1	[−1.0, 3.3]	0.31
Attributable proportion^‡^	0.1	[−0.2, 0.4]	0.40	0.2	[−0.1, 0.6]	0.23
Synergy index^£^	1.1	[0.8, 1.8]	0.43	1.4	[0.7, 2.5]	0.30
Observations		7,687			7,488	

Additive measures calculated using prevalence ratios from Poisson regression models (in Table 3) stratified by race/ethnicity. ^a^Reference group used for comparison: non-Hispanic White participants. ^+^Demographic model: adjusted for age, sex, education. ^$^Behavioral model: adjusted for age, sex, education, smoking status, alcohol consumption, body mass index, exercise, sleep. ^§^ Biomarker model: adjusted for age, sex, education, smoking status, alcohol consumption, body mass index, exercise, sleep, chronic conditions, total cholesterol to high density lipoprotein-cholesterol ratio, glycosylated hemoglobin. ^†^Excess risk = Prevalence ratio (PR) of double exposed–PR of single exposed (minority)–PR of single exposed (CysC) + 1. ^‡^Attributable proportion = [Excess risk due to interaction]/[PR of double exposed]. ^£^Synergy index = [PR of double exposed–1]/[PR of single exposed (minority) + PR of single exposed (CysC)–2]. ^1^CI, confidence interval calculated using the delta method. Values in bold are statistically significant.

Point measures of additive interaction for the Hispanic group were equivalent to those observed for non-Hispanic Black participants (Table 4). Estimates for both groups suggest a potential interaction effect between minoritized race/ethnicity status and high cystatin C. Although some point estimates were not statistically significant, all measures of additive interaction were positive and suggested synergism. We did not observe additive interaction between race/ethnicity and high cystatin C on CIND prevalence.

#### Four-way mediation-interaction decomposition to assess race/ethnicity and cystatin C on dementia prevalence

The decomposition analysis comparing non-Hispanic Black to White participants, adjusting for demographic variables, showed that the mediating effect of cystatin C accounted for 2% (95% CI: −0, 4%) of the observed racial disparity in dementia (Table 5), while the portion attributable to the interaction between exposure and mediator (moderating pathway) accounted for 8% (95% CI: −5, 22%) of the disparity. The decomposition analysis indicated that 91% (95% CI: 78, 105%) of the racial disparity was due to the controlled direct effect of race/ethnicity, pointing that, of course, there are other pathways through which racism operates besides high cystatin C levels (Table 5). These attributable proportions can be interpreted causally if a sufficient set of confounder variables have been identified and no violations of the causal mediation assumptions are present (VanderWeele, 2014).

**TABLE 5:** Decomposition estimates of mediation analysis results, race/ethnicity used as proxy exposure for racism and high serum cystatin C levels (>1.24 mg/L) as mediator.

	Mediation-interaction decomposition^†^							
	Non-Hispanic Black^a^				Hispanic^a^			
Component	Excess relative risk	95% CI	Proportion attributable	95% CI	Excess relative risk	95% CI	Proportion attributable	95% CI
Controlled direct effect	4.00	2.4, 5.6	91.0%	78, 105%	2.88	1.3, 4.4	91.0%	74, 108%
Interaction reference	0.31	−0.2, 0.8	7%	−4, 19%	0.31	−0.2, 0.8	10%	−8, 27%
Interaction mediation	0.06	−0.0, 0.1	1%	−1, 3%	−0.02	−0.0, 0.0	0%	−1, 0%
Pure indirect effect	0.01	−0.0, 0.0	0.0%	−0, 1%	−0.00	−0.0, 0.0	0%	0, 0%
Total effect	4.38	2.9, 5.9	100%		3.17	1.8, 4.6	100%	
**Overall proportion**								
Due to mediation			2%	−0, 4%			−1%	−2, 0%
Due to interaction			8%	−5, 22%			9%	−7, 26%

Models allow for exposure-mediator interaction effects. Estimates presented by race/ethnic groups. ^a^Reference group used for comparison: non-Hispanic White participants. ^†^Demographic model: adjusted for age, sex, education.

For the Hispanic group, the demographic-adjusted decomposition analysis showed similar interaction results to that of the non-Hispanic Black group. Among Hispanic participants, the mediating effect of cystatin C in the racial disparity was virtually zero, while the portion attributable to the interaction accounted for 9% (95% CI: −7, 26%) of the racial disparity and the direct effect for 91% (95% CI: 74, 108%) (Table 5). We did not estimate attributable fractions for models controlling for behavioral risk factors, or other biomarkers, as the mediating effect of cystatin C was negligible for both minoritized racial groups with further adjustment.

### Sensitivity analyses

We re-estimated measures of additive interaction to test whether the omission of the genetic influence of *APOE*-ε*4* on dementia biased our mediation decomposition results. After adjusting for *APOE*-ε*4*, prevalence ratios of dementia for the double and single exposed groups (Supplementary Table 5) were consistent to our demographic adjusted models without the inclusion of *APOE*-ε*4* (Table 4).

To assess robustness of our findings to potential dementia misclassification, we repeated our analyses using Power’s two-level dementia outcome. In the demographic-adjusted model, among non-Hispanic Black participants with high cystatin C (double exposed group), the prevalence of dementia was 2.6 (95% CI: 2.0, 3.3) times higher than for non-Hispanic White participants with low cystatin C (unexposed group) (Supplementary Table 6A). For Hispanic participants, using Power’s algorithm did not show an association between Hispanic ethnicity, high cystatin C levels, and dementia (Supplementary Table 6B). Additionally, measures of interaction in the additive scale were close to the null value and non-significant for both minoritized social groups (Supplementary Table 7).

## Discussion

In this large and diverse cross-sectional analysis of older adults in the United States, high levels of serum cystatin C (>1.24 mg/L) were associated with elevated prevalence of cognitive impairment non-dementia and dementia relative to normal cognition. The association between high cystatin C and dementia was suggestive of super-additivity for minoritized racial groups, but the confidence intervals for the additive measures of interaction were imprecise. We found that 8% of the disparity in prevalent dementia between non-Hispanic Black and non-Hispanic White participants was due to the interaction between race and high cystatin C levels, while 2% was mediated through cystatin C. Altogether, these results suggest that, among older adults who are racialized as non-Hispanic Black or Hispanic, the effect of high cystatin C levels on dementia prevalence is greater than expected had these individuals been racialized and treated as non-Hispanic White (Phelan and Link, 2015; Asad, 2018; Graetz et al., 2022). However, the largest proportion of the observed disparity could be thus attributable to other pathways through which racism operates besides cystatin C (Graetz et al., 2022).

Three observational studies previously documented an association between elevated cystatin C levels and adverse cognitive health outcomes (Sarnak et al., 2008; Yaffe et al., 2008; Slinin et al., 2015). The Aging Brain Cohort (ABC) study, another large racially diverse study conducted in the United States, found that individuals with cystatin C serum levels >1.25 mg/L had 1.92 times higher odds of 7-year incident cognitive impairment with respect to those with cystatin C levels <1.0 mg/L (Yaffe et al., 2008). The ABC study limited race-dependent analysis to a multiplicative interaction; however, we expanded on their analysis by testing additive interactions between cystatin C and race/ethnicity. The Cardiovascular Health Study, a community-based longitudinal cohort of older adults (aged ≥65) with a representative sample of non-Hispanic Black participants, estimated a 1.39 greater hazard of cognitive impairment among those with cystatin C levels >1.16 vs. ≤0.90 mg/L (Sarnak et al., 2008). This study did not explore an interaction between race and cystatin C. Lastly, a prospective cohort of women aged >65 in the United States showed that cystatin C levels of 1.15−2.37 mg/L (vs. 0.61−0.91 mg/L) were associated with 1.4 times higher odds of 10-year incident cognitive impairment, after adjusting for race and age (Slinin et al., 2015). Each of these three studies linked elevated cystatin C levels to worse cognitive status, and all concluded that the effect of cystatin C levels on cognitive status was homogeneous across racial/ethnic groups. Our analysis builds on these findings, by suggesting that the effect of cystatin C on dementia differs based on whether older adults are racialized as non-Hispanic Black or Hispanic rather than non-Hispanic White.

Of the previous studies, only the ABC study explored an interaction effect between race and cystatin C levels (Yaffe et al., 2008). Interaction effects are of public health importance because they illustrate which social groups are at most risk for adverse health events and could benefit from early interventions. However, statistical tests for interaction are scale dependent and test results may vary depending on the scale in which interaction effects are assessed—additive vs. multiplicative (Greenland, 1993, 2015). In the ABC study, researchers tested for a multiplicative interaction effect between race and cystatin C, concluding that no interaction was present (Yaffe et al., 2008). Nonetheless, epidemiologists prefer estimating interaction effects in the additive scale (Greenland, 2009). Our mediation-interaction approach allowed us to quantify two important components of the racial disparity: (1) the proportion due to the interaction between race/ethnicity and cystatin C (moderating pathway), and (2) the proportion due to the mediating effect of cystatin C.

We found that the effect of cystatin C on dementia may be modified by minoritized racial status, as evidenced by two of our measures: the excess risk due to interaction (a measure of additive interaction) and the proportion due to the interaction (a decomposition component of the mediation analysis). We observed that for minoritized participants with high levels of cystatin C (double exposed) the prevalence of dementia was higher than that of non-Hispanic White participants with low cystatin C (unexposed) or participants with either one of the two factors alone (single exposed). These findings held independently of demographic, behavioral, and other biomarkers and chronic conditions. Mediation-interaction analysis results indicated that the interaction effect was responsible for explaining at least 9% percent of the racial disparity in prevalent dementia across both minoritized groups. The mediating effect of cystatin C only explained a small portion of the racial disparity for non-Hispanic Black participants and virtually none for Hispanic participants. Our interaction results suggest that when comparing Hispanic to non-Hispanic White participants, the association between elevated cystatin C on prevalent dementia was of lesser magnitude than when comparing the non-Hispanic Black participants to their non-Hispanic White counterparts. We attribute these findings to Hispanic participants being of younger age, having less chronic health conditions, and comparable average blood levels of cystatin C than their non-Hispanic White counterparts in our study sample. The lack of mediation effect between Hispanic participants relative to non-Hispanic White participants may be because disparities in high levels of cystatin C across both groups are accounted for by covariates such as behavioral risk factors and other biomarkers, and because it is unlikely that cystatin C is the only pathway implicated in the production of racial disparities in prevalent dementia. Future research should focus on incorporating multiple mediators to better understand which deteriorated physiological systems are more likely to mediate racial disparities in cognitive aging.

Research has demonstrated that cystatin C concentrations in blood are influenced by genetic polymorphisms of the *CST3* gene (Maniwa et al., 2020; Ding et al., 2021). For instance, a mutation at the + 148 position in *CST3* gene alters the secretion of cystatin C, leading to an imbalance between higher intracellular levels of cystatin C and concomitant increase of protease activity, but lower extracellular levels (Maniwa et al., 2020). These polymorphisms have been associated with structural brain changes such as periventricular hyperintensity, deep subcortical white matter hyperintensity, and large-artery atherosclerotic stroke, independently of participants’ kidney function (Maniwa et al., 2020; Ding et al., 2021). The interplay between *CST3* gene polymorphs and the *APOE-ε4* allele is less understood. But at least two case-control studies have explored the relationship between *CST3* polymorphism and *APOE-ε4* allele in Alzheimer’s patients of European ancestry, (Finckh et al., 2000; Beyer et al., 2001) one concluding a synergistic association between *APOE-ε4* allele and *CST3* polymorphism on dementia risk (Beyer et al., 2001). And another one, concluding that the association of these two genes on dementia were independent (Finckh et al., 2000). However, studies exploring this synergistic effect among participants of non-European ancestry are lacking. Our sensitivity models suggest that the association between elevated circulating levels of cystatin C and the prevalence of dementia among non-Hispanic Black and Hispanic adults was independent of *APOE-ε4* allele carrier status, since non-Hispanic Black and Hispanic participants with elevated cystatin C levels had higher prevalence of dementia than their non-Hispanic White counterparts after adjustment by *APOE-ε4* allele carrier status. But the association between elevated cystatin C and dementia was null for non-Hispanic White adults when *APOE-ε4* was added to the model. These findings suggest that the higher prevalence of dementia conveyed by elevated cystatin C in non-Hispanic White Americans may be confounded by *APOE-ε4* allele status. Future studies should explore the relationship between *CST3* gene polymorphism, *APOE-ε4* and dementia risk in participants of non-European and European ancestry to better characterize the role of genetic susceptibility, serum cystatin C levels, and dementia risk among diverse populations in the United States. Additionally, because dementia classification algorithms may have differential sensitivity and specificity performance across racial/ethnic groups, we also conducted our analyses using an alternative dementia classification method known to have similar sensitivity and specificity by racial groups (Gianattasio et al., 2020). The use of Power’s algorithm for dementia classification dramatically attenuated the point estimates for our double exposed subgroups (non-Hispanic Black and Hispanic participants with high cystatin C) and hence their measures of additive interaction. Because the Power’s algorithm only classifies participants into two categories (dementia vs. normal cognition), a sizable proportion of participants with cognitive impairment non-dementia (84%) or dementia (40.4%) according to the Langa-Weir algorithm may be classified as cognitively normal by the Power’s algorithm, which could attenuate any association between our variables of interest and dementia. In fact, our results found a super-additive effect between race/ethnicity and high cystatin C levels in participants with dementia but not with CIND which may help explain why our results using Power’s algorithm were indicative of no interaction in the additive scale.

This study’s strengths include quantification of the effect of cystatin C levels on two distinct cognitive outcomes in a large sample of non-Hispanic Black, Hispanic, and non-Hispanic White adults. We had rich data on several confounders, including a genetic risk factor for dementia and we conducted a sensitivity analysis to estimate the robustness of our results to potential misclassification of the outcome. Furthermore, we employed a novel statistical approach to accommodate effects due to interaction and mediation into a single framework. Limitations are that our data were cross-sectional, and thus subject to reverse causation and prevalence-incidence bias. Serum cystatin C levels may not be representative of cerebrospinal fluid concentrations, with limited direct relation to underlying neuropathological changes related to dementia (Sundelöf et al., 2008). In fact, cerebrospinal fluid concentrations of cystatin C are approximately 5.5 times higher than serum circulating levels, indicating that the microenvironment of the central nervous system might exert local influence independently of serum levels (Maniwa et al., 2020). Our lack of repeated measures of cystatin C is an important limitation, and further studies should explore how changes in cystatin C levels may differ by race/ethnicity and differentially affect cognitive performance over time. Another important limitation is that our study only included educational attainment as a unique proxy measure for socioeconomic status. Therefore, our results may be susceptible to residual confounding since other socio-economic measures such as wealth, occupation, and neighborhood characteristics may be associated with disparities in prevalent dementia among the three different racialized social groups in our study. Finally, our models did not adjust for glomerular filtration rate, and the depuration of circulating cystatin C may be affected by kidney functioning; (Peralta et al., 2011a,b) therefore, we do not know to what extent our results may be susceptible to residual confounding effects. However, the ABC study showed that effect of cystatin C levels on cognition was independent of the effect of glomerular filtration rate (Yaffe et al., 2008).

In conclusion, we observed that elevated serum cystatin C was associated with 1.2 times higher prevalence of dementia in a large, diverse sample of middle-aged and older US adults. The effect of cystatin C on prevalent dementia may be moderated by race/ethnicity, indicating that racialization affects not only the distribution of serum cystatin C across minoritized racial groups, but also the strength of association between the biomarker and dementia. Our findings add to the increasing body of epidemiological evidence linking elevated circulating levels of cystatin C to adverse cognitive aging, elucidating potential shared biological mechanisms implicated in early stages of renal disease and vascular forms of dementia. Furthermore, we identified that non-Hispanic Black and Hispanic individuals with high levels of cystatin C are at increased risk for dementia. Public health efforts should focus on improving renal health outcomes in these vulnerable populations to ameliorate racial disparities in dementia. Future approaches should identify the role of other physiological pathways involved in the production of racial disparities in dementia. Our results provide compelling evidence that cystatin C is associated with adverse brain health, and this effect is larger than expected for individuals who are racialized as minorities. By implementing a novel methodological approach for causal mediation analysis, we illustrated how physiological biomarkers can be incorporated in the study of racial disparities in dementia.

## Data availability statement

The current study is a secondary data analysis of existing data that are already available in a publicly accessible data repository. The data presented in this study were accessed from the http://hrs.isr.umich.edu/data-products data repository, Health Data: 2016 Biomarker Data release date November 2020 (Early v1.0).

## Ethics statement

The studies was involving human participants were reviewed and approved by the University of Michigan Institutional Review Board. The patients/participants provided their written informed consent to participate in this study.

## Author contributions

CH, KB, and EW contributed to the conception and design of the study. CH organized the database and performed the statistical analysis. MF performed the code and manuscript revision. LK, MH, MZ, and BM contributed to the manuscript revision, read, and approved the submitted version. All authors contributed to the article and approved the submitted version.

## Footnotes

1
https://hrs.isr.umich.edu/data-products

2 https://dbgap.ncbi.nlm.nih.gov; phs000428.v2.p2

3
https://github.com/bakulskilab
